# Serum HMGB1 as a Potential Biomarker for Patients with Asbestos-Related Diseases

**DOI:** 10.1155/2017/5756102

**Published:** 2017-03-01

**Authors:** Shibo Ying, Zhaoqiang Jiang, Xianglei He, Min Yu, Riping Chen, Junqiang Chen, Guoqing Ru, Yuan Chen, Wanyuan Chen, Lijin Zhu, Tao Li, Yixiao Zhang, Xinnian Guo, Xianhong Yin, Xing Zhang, Jianlin Lou

**Affiliations:** ^1^Institute of Occupational Diseases, Zhejiang Academy of Medical Sciences, Hangzhou 310013, China; ^2^Department of Pathology, Zhejiang Provincial People's Hospital, Hangzhou, Zhejiang 310014, China; ^3^Jiading District Center for Disease Control and Prevention, Shanghai 201800, China

## Abstract

High-mobility group box 1 (HMGB1) functions as a proinflammatory cytokine and is one of the most intriguing molecules in inflammatory disorders and cancers. Notably, HMGB1 is a potential therapeutic target and novel biomarker in related diseases. However, the diagnostic value of HMGB1 for benign and malignant asbestos-related diseases (ARDs) remains unclear. In this work, we detected preoperative serum HMGB1 levels in Chinese asbestos-exposed (AE) and ARDs populations and further evaluated the diagnostic value of HMGB1 in patients with certain types of ARDs, including those with pleural plaques, asbestosis, or malignant mesothelioma (MM). The experimental data presented that the serum level of HMGB1 was significantly elevated in AE and ARDs subjects. Our findings indicated that serum HMGB1 is a sensitive and specific biomarker for discriminating asbestosis- and MM-affected individuals from healthy or AE individuals. In addition, serum matrix metalloproteinases 2 and 9 are not correlated with HMGB1 in ARDs. Thus, our study provides supporting evidence for HMGB1 as a potential biomarker either for the clinical diagnosis of high-risk AE cohorts or for evaluating ARDs.

## 1. Introduction

Asbestos exposure and asbestos-related diseases (ARDs) are a global health issue. The epidemic of ARDs will likely continue to increase for at least another decade in most industrialized countries and for several decades in industrializing countries before reaching its peak [1]. Globally, occupational exposure to asbestos causes an estimated 107,000 deaths each year. Most deaths result from certain types of ARDs, such as asbestos-related lung cancer, asbestosis, and mesothelioma [1]. In China, the estimated number of occupationally exposed workers may be over one million, while the prevalence and incidence of ARDs as well as the mortality from ARDs are expected to be substantial and are likely to increase [2]. Malignant ARDs continue to present challenges in the arenas of occupational medicine, public health, and clinical research and practice [3]. These serious diseases require early prevention, recognition, and treatment in the future.

High-mobility group box 1 (HMGB1) is a member of the high-mobility group superfamily, which exists ubiquitously in the nuclei and cytosols of mammalian cells [4]. HMGB1 is a DNA chaperone primarily found in the nucleus, where it stabilizes nucleosomes and contributes to DNA transcription, replication and recombination [4–6]. Under certain physiological or pathological conditions, HMGB1 can translocate from the nucleus to the cytosol and then be secreted into the extracellular environment [4, 6]. HMGB1 functions as a proinflammatory cytokine due to its active secretion by innate immune cells, such as neutrophils, monocytes, macrophages, and several tumor cells, but it can also be released passively during cell injury and death [7]. Recent in vitro experimental evidence showed that asbestos fibers could induce programmed necrosis and inflammation in primary human mesothelial cells [8]. Upon asbestos exposure, HMGB1 translocates from the nucleus to the cytosol and is released into the cell culture medium [8]. Subsequently, HMGB1 release induces macrophages to secrete tumor necrosis factor-*α* (TNF-*α*), which protects mesothelial cells from asbestos-induced cell death and triggers a chronic inflammatory response [8]. Chronic inflammation is also the hallmark of asbestos deposition in tissue and contributes to its carcinogenesis [9].

As a possible mechanism in asbestos-induced inflammation, HMGB1 may be secreted into the stroma and subsequently makes its way into the systemic circulation [9]. Recent clinical studies have shown that HMGB1 is a potential diagnostic or prognostic biomarker in a variety of inflammatory disorders [10–12] and cancers [13–19]. One group from the USA provided the first evidence that HMGB1 was highly expressed in both tissues and sera of mesothelioma patients [20]. In a following report with similar results based on the exposure of Japanese subjects to asbestos, the HMGB1 protein levels in the sera from patients with diffuse malignant peritoneal mesothelioma were also reported to be significantly higher than those of nonmesothelioma subjects with a history of exposure to asbestos [21]. Serum HMGB1 was also a prognostic marker for Japanese patients with malignant pleural mesothelioma (MPM) [22]. However, the clinical importance of HMGB1 for other nonmalignant ARDs is still unknown. Little data is available for serum HMGB1 levels from a Chinese asbestos-exposed (AE) population.

In the present study, we quantified serum HMGB1 levels in a Chinese AE population and further evaluated the diagnostic value of HMGB1 in patients with nonmalignant and malignant ARDs, including pleural plaques (PP), asbestosis, and malignant mesothelioma (MM). We provide evidence supporting the hypothesis that HMGB1 could be a candidate biomarker in clinical diagnosis for MM and asbestosis.

## 2. Materials and Methods

This study was conducted in accordance with the Declaration of Helsinki and was approved by the Medical Ethics Committee of Zhejiang Academy of Medical Sciences.

### 2.1. Study Population and Data Collection

A total of 497 subjects were recruited from several small neighboring towns with a long history of asbestos industry during the years 1960 to 2012 in Southeast China [23]. Chest X-rays were taken for all of the subjects. Nonmalignant asbestos-related diseases were diagnosed according to the American Thoracic Society (ATS) criteria [24]. MPM was diagnosed by at least two independent pathologists according to the Guidelines for Pathologic Diagnosis of Malignant Mesothelioma proposed by the International Mesothelioma Interest Group [25]. A standardized questionnaire focused on occupational history was taken during a face-to-face interview. The contents of questionnaires included age, gender, smoking and drinking habits, occupation, and jobs entailing past asbestos exposure. For those who were occupationally exposed to asbestos, exposure duration was measured as the interval of years between the start and the end of AE jobs. Potential exposure levels to asbestos were estimated according to the approach in our previous report [26]. Briefly, we retrieved periodic data of total dust concentrations in different workshops/manufactories available from 1984 to 2010 in this area. The median dust concentrations were 0.7 mg/m^3^ in a household textile workshop (small-scale) and 5.9 mg/m^3^ in a textile manufactory (large-scale). Based on the corresponding median dust concentration, potential exposure levels were calculated (in mg/m^3^  ×  years) when applicable.

### 2.2. Grouping Sets

The study subjects were divided into six groups: (1) a healthy control group that consisted of 71 healthy subjects without any known history of asbestos exposure and with normal chest X-ray examination results; (2) a group of 170 subjects occupationally exposed to asbestos for less than 10 years, but with normal chest X-ray examination results; (3) a group of 129 subjects occupationally exposed to asbestos for over 10 years, but with normal chest X-ray examination results; (4) a group of 81 subjects with PP found in their chests; (5) a group of 31 patients diagnosed with asbestosis; and (6) a group of 15 patients diagnosed with MPM.

### 2.3. Detection Using Blood Samples

Blood samples were collected using standard blood procurement protocols from the enrolled subjects who provided written informed consent for participation in this approved study. Five milliliters of blood was collected from the antecubital vein. After clotting, these samples were centrifuged at 4000 rpm for 5 min. The subsequent separated sera were stored at −80°C until detection assays for HMGB1 and matrix metalloproteinases (MMPs) were performed. The concentrations of HMGB1, MMP2, and MMP9 in the sera were measured using enzyme-linked immunosorbent assays (ELISAs) according to the manufacturer's instructions (Cloud-Clone Corp., Houston, TX, USA). All assays were performed in duplicate.

### 2.4. Statistical Analysis

Statistical analyses were performed using GraphPad Prism 5 software (GraphPad Software Inc., La Jolla, CA, USA) or SPSS Statistics 17.0 (SPSS Inc., Chicago, IL, USA). Qualitative data are reported as frequencies and proportion distributions. The chi-square test was used for comparison of the proportions between groups, and the significance level was adjusted using the Bonferroni method. Quantitative data with nonnormal distributions are presented as medians and interquartile ranges. A Kruskal-Wallis test followed by Dunn's multiple comparisons test was conducted to compare the median values between groups. Statistical significance was defined as a two-sided *P* < 0.05. Univariate and multivariate linear regression analyses were fit to explore predictors of HMGB1. The receiver operating curve (ROC) method was conducted for differentiating ARDs and AE groups from the control group. Areas under ROC curves (AUCs) were presented with 95% confidence intervals (CIs). An AUC of 1.0 indicates perfect discrimination, whereas an area with a confidence interval of 0.5 indicates that the test discriminates no better than chance. The optimum statistical cut-off value was selected when Youden's index (sensitivity + specificity-1) reached the maximum value. The relationships among the correlations between HMGB1, MMP2, and MMP9 were identified using Spearman's rank correlation coefficient (*r*_*s*_).

## 3. Results 

### 3.1. Characteristics of the AE and ARD Populations

The demographic and occupational characteristics of subjects, including age, gender, smoker, drinker distribution, potential asbestos exposure level, and exposure duration in study groups, are presented in Table 1. The mean ages among these groups were significantly different (*χ*^2^ = 13.37, *P* = 0.02). Subjects with asbestosis were significantly older (median age, 73 years) than the AE < 10 years group (median age, 66 years; *P* < 0.05) and the MPM group (median age, 66 years; *P* < 0.05). The gender distributions were significantly different among these groups (*χ*^2^ = 50.15, *P* < 0.01), and the AE, PP and asbestosis groups had larger proportions of female subjects than the control group. The reason for the marked proportional difference is that most asbestos hand-spinning workers are female and tend to be more heavily exposed than males [23]. Both the potential AE level and the exposure duration were significantly different among these groups (*χ*^2^ = 83.99, *P* < 0.0001; *χ*^2^ = 1223.19, *P* < 0.0001). Of these three ARDs groups, the PP group had the highest potential AE level (50.2 mg/m^3^) every year. In contrast, MPM patients, the only malignant ARDs group in this study, had the lowest potential AE level (11.0 mg/m^3^). Furthermore, the mean asbestos exposure durations in both the PP and asbestosis groups were 10 years. The MPM group had a longer exposure duration (14 years) than these two nonmalignant groups.

### 3.2. Serum HMGB1 as a Potential Biomarker for ARDs

**Figure 1:**
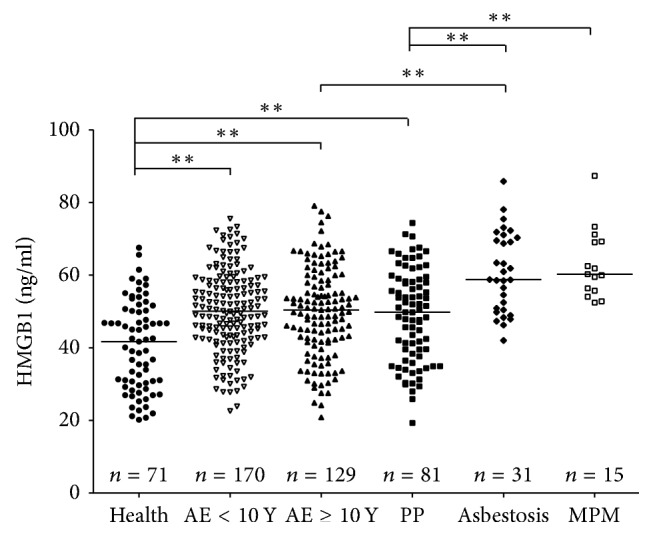
HMGB1 levels in serum from individuals with pleural plaques (PP), asbestosis, and MPM, exposed to asbestos and healthy controls. ELISAs shown were performed in parallel and blindly. Bars show the median of HMGB1 levels. Statistical significance was defined as two-sided ^*∗∗*^*p* < 0.01.

To investigate whether serum HMGB1 functions as a potential biomarker for identifying AE/ARD individuals or cohorts, we tested HMGB1 levels in sera from all six groups. As shown in Figure 1, the median levels of serum HMGB1 in the AE groups, 50.06 ng/mL (AE < 10 years) and 50.42 ng/mL (AE ≥ 10 years), were similar. Both of these values were significantly higher than the median level of 41.68 ng/mL observed in healthy controls. However, no significant differences in HMGB1 levels were observed between the two groups with different exposure durations (AE < 10 and AE ≥ 10 years). These results strongly suggest that HMGB1 may be a useful biomarker for evaluating AE individuals.

The serum HMGB1 levels in the PP group (median level: 49.77 ng/mL) were significantly higher than that of the control group but were approximately equal to that of the two AE groups. HMGB1 levels in the asbestosis group (median level: 58.77 ng/mL) were significantly higher than those of the control group, the two AE groups, and the PP group. Similarly, HMGB1 levels in the MPM group (median level: 60.23 ng/mL) were also significantly higher than those of the control, AE, and PP groups. Nevertheless, there was no significant difference in HMGB1 levels between the asbestosis and MPM groups. Our findings indicated that HMGB1 is a potential biomarker to screen certain subsets of patients with ARDs (asbestosis and MPM) from AE and healthy individuals. However, the HMGB1 serum level is not useful for discriminating patients with MM from those with asbestosis.

### 3.3. Multiple Factors May Influence the Serum Level of HMGB1

To test whether the predicted factors, including age, gender, smoking, asbestos exposure duration, and potential exposure levels, influence HMGB1 serum levels, we performed univariate and multivariate linear regression analyses. As shown in Table 2, the regression coefficients, standard errors of coefficients, and *P* values were analyzed by linear regression. In the results of univariate regression analysis, dummy variables of PP, AE, asbestosis, and MPM groups showed significant influences on the serum level of HMGB1, with a positive coefficient. In addition, the results from the multivariate regression analysis revealed that dummy variables of AE, PP, asbestosis and MPM groups had significantly positive influences on serum HMGB1 levels. However, we did not find significant differences between different groups of potential exposure levels (*χ*^2^ = 1.70, *P* = 0.43) and exposure duration (*χ*^2^ = 0.34, *P* = 0.84). Taken together, these findings support the notion that HMGB1 may be a valuable independent index to differentiate AE and ADRs patients from healthy individuals.

### 3.4. HMGB1 Is Sensitive and Specific for Discriminating ARDs from Controls

**Figure 2:**
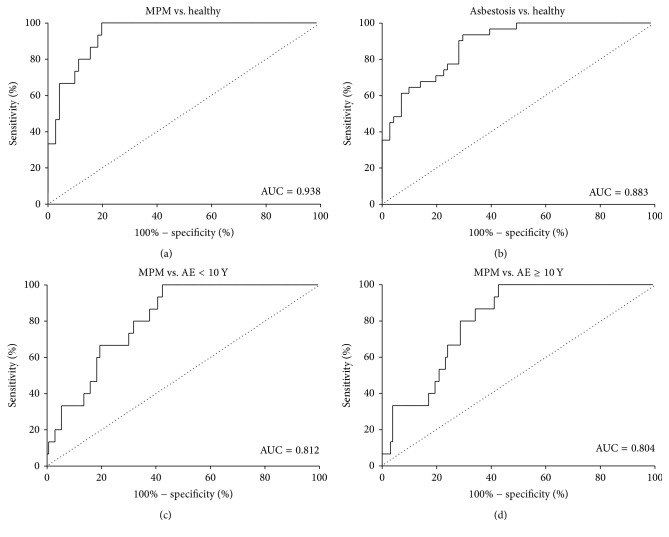
ROC curves of HMGB1 for distinguishing patients with asbestos-related diseases (ADRs) from AE individuals or healthy controls. Four ROCs with high AUC (>0.800) were selected and shown in panels (a), (b), (c), and (d). ROC: receive operation curve; AE: asbestos-exposed; AUC: area under the curve; vs.: versus; Y: years. See details in text.

To further evaluate the diagnostic value of HMGB1 to discriminate ARDs from AE individuals and healthy controls, we calculated the sensitivity and specificity of HMGB1 as a potential biomarker. According to the sizes of the AUC, the comparisons were subsequently ranked (Table 3). Due to the considerably high diagnostic value, four ROCs with high AUC (>0.800) were selected and are shown in Figure 2. The AUC for HMGB1 values was 0.94 (95% confidence interval: 0.89–1.03) for distinguishing MPM from healthy controls. An HMGB1 level of 52.16 ng/mL was determined as the best cut-off value with a sensitivity of 100% and a specificity of 80.28%. The AUCs for HMGB1 values to distinguish MPM from AE < 10 years and ≥10 years were 0.81 (95% CI: 0.73–0.90) and 0.80 (95% CI: 0.72–0.89), respectively.

The best cut-off value was 52.29 ng/mL (sensitivity: 100%, specificity: 57.65%) for distinguishing MPM from AE < 10 years. Similarly, the optimal cut-off value was 52.39 ng/mL (sensitivity: 100%, specificity: 57.36%) for distinguishing MPM from AE ≥ 10 years. In addition, the AUCs for HMGB1 values to distinguish asbestosis from subjects of AE < 10 years, AE ≥ 10 years and healthy controls were 0.74 (95% CI: 0.66–0.83), 0.74 (95% CI: 0.64–0.83), and 0.88 (95% CI: 0.82–0.95), respectively (Table 3, Figure 2). Overall, HMGB1 is a good diagnostic index to identify MPM patients among AE and healthy cohorts. In addition, HMGB1 is also a valuable biomarker in distinguishing patients with asbestosis from healthy control subjects.

### 3.5. Secreted MMP2/9 Are Not Correlated with HMGB1 in ARDs

MMPs are expressed in a variety of connective tissue types and proinflammatory cells and are considered as promising therapeutic targets due to their strong involvement in key pathological events. Among the MMPs, both MMP2 and MMP9 function as secreted gelatinases, which are responsible for tissue remodeling and the degradation of extracellular matrix proteins, such as collagen and gelatin. HMGB1 has been reported to mediate MMP2 and MMP9 expression in some inflammatory disorders and cancers [27–31]. To test whether their levels correlated with HMGB1, we further measured the concentrations of MMP2 and MMP9 in sera from the studied population. Serum levels of MMP2 and MMP9 are shown in Supplementary Figures S1 and S2 (see Supplementary Materials available online at https://doi.org/10.1155/2017/5756102). We found that MMP2 was positively correlated with serum HMGB1 levels in subjects with AE < 10 years (*r*_*s*_ = 0.2215, *P* = 0.0037) and AE ≥ 10 years (*r*_*s*_ = 0.2345, *P* = 0.0075). Similarly, MMP9 was positively correlated with serum HMGB1 levels in healthy controls (*r*_*s*_ = 0.2580, *P* = 0.0298) and AE < 10 years (*r*_*s*_ = 0.2721, *P* = 0.0003). Interestingly, both MMP2 and MMP9 serum levels were not correlated with HMGB1 in ARDs, including PP, asbestosis and MPM (Table 4). MMP2 and MMP9 were correlated with each other in all groups, except for the asbestosis group (Table 4). Although it was confirmed that MMP2 and MMP9 expression levels were closely linked with HMGB1-mediated mechanisms, our findings indicated that serum MMP2/9 levels may not represent helpful supplementary indexes for HMGB1 in the clinical diagnosis of ARDs.

## 4. Discussion

In East Asia, ARDs associated with occupational and environmental asbestos exposure and related products contaminated with asbestos have gathered continuous public attention. This study provided exhaustive data of one proinflammatory cytokine, HMGB1, from Chinese populations with AE and ARDs. Our results indicated that there are associations between serum HMGB1 levels and asbestos and different ARDs. Most ARDs are diagnosed in those who have experienced occupational or environmental asbestos exposure. It is possible that the serum level of HMGB1 is associated with previous cumulative exposure to asbestos, which in turn may be strongly linked to the severity of inflammatory disorders. Increasing clinical evidence indicates that extracellular HMGB1 contributes to inflammatory disorders and cancer development [5, 6, 32]. As a potential biomarker, our results strongly support the clinical application value of HMGB1 in screening for severe ARDs. To our knowledge, this study is the first to report serum HMGB1 levels from Chinese patients with benign ARDs and MPM.

As an alarmin, HMGB1 signals cell damage in response to injury and inflammation [5]. In agreement with previous studies in a variety of cell lines and animal model systems, our results support the notion that circulating HMGB1 is caused by exposure to asbestos fibers. HMGB1 production in mesothelioma cells in vitro, such as H2052 (epithelioid) and H28 (sarcomatoid), produced higher HMGB1 protein levels than that of a normal human mesothelial cell line [22]. Similarly, in both mice and hamsters injected with asbestos fibers, HMGB1 was observed in the nuclei, cytoplasm, and extracellular spaces of mesothelial and inflammatory cells around asbestos deposits in vivo [8]. Serum levels of HMGB1 persisted long-term after exposure to crocidolite fibers but not to chrysotile in asbestos-injected mice. However, prolonged chrysotile exposure may induce sustained serum levels of HMGB1 [33]. Remarkably, chrysotile is one type of asbestos that accounts for approximately 90% of asbestos used commercially worldwide [34] and is also the main raw material for asbestos product manufacturers from Southeast China in our study [23]. Pulmonary receptor for advanced glycation end-products (RAGE), a well-characterized cell surface receptor of HMGB1, was down-regulated after asbestos injury in a mouse model of pulmonary fibrosis [35]. In most healthy tissues, RAGE is expressed at low to undetectable levels. However, its expression in the lung is very high under normal conditions and is depleted in fibrotic disease states, which may have the protective effects of accepting HMGB1 signaling, thus causing chronic inflammation [36]. This result suggested that substantial HMGB1 is to the extracellular space during asbestos-induced pulmonary fibrosis. Combined with these previous findings, our results show that HMGB1 may be a key inflammatory mediator in ARDs. Novel strategies targeting HMGB1, such as glycyrrhizin [37], gabexate mesilate [37], ethyl pyruvate [38, 39], and PPAR ligands [40] that interfere with asbestos-mediated inflammation, may prevent or delay ARDs onset and relieve the progress of malignant ARDs.

The possibility that HMGB1 may be a blood biomarker for MM or other ADRs is of particular interest. Human MM is an aggressive and highly lethal cancer predominantly caused by chronic exposure to asbestos and erionite. The prognosis for this cancer is generally poor due to late-stage diagnosis and resistance to current conventional therapies [41]. One study from Japan reported that the diagnostic sensitivity of serum HMGB1 for MPM measured on an ROC curve was not high (34.4%), but its specificity and positive predictive value were extremely high (100% and 100%, resp.) [22]. Similarly, one research group from the USA also reported that the HMGB1 concentrations in the sera of patients with MM were significantly higher than those in sera from healthy controls [20, 42]. Furthermore, they determined that hyperacetylated HMGB1, a specific subset of HMGB1 molecules, reliably distinguished MM patients from individuals who were occupationally exposed to asbestos with 100% sensitivity and specificity [42]. Our results also support the notion that HMGB1 is a potential biomarker to discriminate MM patients from asbestos-exposed patients with benign ARDs (i.e., PP) or healthy individuals. However, there are no previous statistics of total serum HMGB1 levels to perform a comparison between patients with asbestosis and MM. To our knowledge, this study is the first to report serum HMGB1 levels from the patients with asbestosis. Although total HMGB1 levels are sensitive and specific to discriminate ARDs from unexposed individuals, the very low AUC of 0.56 observed when comparing MPM patients with asbestosis individuals would limit its clinical utility for identifying different types of ARDs patients among large cohorts of AE or healthy individuals. Notably, we recently reported a high incidence of peritoneal mesothelioma in relatively young women (mean age of 52.4 years) from Southeast China [23]. Furthermore, epidemiologic investigations have shown that local women who worked at asbestos plants also performed hand spinning with their families, including children, at home. Other studies have indicated that improper storage of asbestos products also may lead to household or environmental exposure [23, 43, 44]. From our perspective, HMGB1 may be a suitable blood biomarker to monitor occupational workers and their families who have a history of residential exposure to asbestos, but accurately discriminating MM and asbestosis requires further investigation. Nevertheless, the combination of serum HMGB1 and radiographic findings should be helpful to stratify the risk of MM in AE populations.

Our work also found that the protein levels of both MMP2 and MMP9 were not correlated with levels of HMGB1 in sera of patients with asbestosis or MM. It is well known that MMPs are involved in the process of metastasis formation and that their overexpression correlates with the processes of tumor cell invasion and metastasis in human cancers by degrading the extracellular matrix [45, 46]. Among MMPs, the activation of MMP2 and MMP9 is also associated with the pathogenesis of airway inflammation and remodeling [47]. Extracellular HMGB1 may activate the RAGE-Ras-MAPK pathway, which results in MMP2 and MMP9 expression [27]. For example, in non-small-cell lung cancer (NSCLC) cells, HMGB1 overexpression resulted in increased MMP9 expression, which was related to a higher metastasis rate [27, 30]. HMGB1 may also trigger MMP9 upregulation in neurons and astrocytes in mouse brains after cerebral ischemia [28]. In patients with ischemic stroke, increased plasma levels of MMP9 and HMGB1 are associated with a poor functional outcome and are significantly correlated with each other [29]. In our study of ADRs, serum MMP2 and MMP9 levels were not significantly positively correlated with HMGB1 levels; therefore, the diagnostic value of serum MMP2 and MMP9 was not useful (Table 4). Another study also supports this notion [48]. Combining these results, it is likely that extracellular HMGB1 may induce intracellular MMP2 and MMP9 expression in local tissue cells but not their secretions into the blood. Hence, the underlying mechanism of secreted MMPs involving their release and circulation is complex in ADRs and remains to be elucidated in the future.

To summarize, serum HMGB1 levels were high in the AE population and particularly high in asbestosis and MM patients. HMGB1 may be a useful clinical biomarker for screening severe ARDs, and it is of potential diagnostic value for evaluating high-risk AE cohorts. This evidence also suggests that HMGB1 is a potential therapeutic target in ARDs.

## 5. Conclusions

To our knowledge, this is the first clinical study describing serum HMGB1 levels in asbestos-exposed and ARDs populations in China. Our study demonstrated that the expression of HMGB1 was significantly elevated in AE and ARDs subjects. For clinical diagnosis, these results indicated that serum HMGB1 is a sensitive and specific biomarker to discriminate asbestosis and MM from healthy or AE individuals. The findings in this work may also provide new insights into the molecular mechanisms of the progression and prognosis of ADRs and may lead to new approaches for effective diagnosis and therapy.

## Supplementary Material

Figure S1: MMP2 levels in serum from individuals with pleural plaques (PP), asbestosis, MPM, exposed to asbestos, and healthy controls. Figure S2: MMP9 levels in serum from individuals with pleural plaques (PP), asbestosis, MPM, exposed to asbestos, and healthy controls.

## Figures and Tables

**Table 1:** Basic characteristics of the subjects.

	Control (*n* = 71)	AE < 10 Y (*n* = 170)	AE ≥ 10 Y (*n* = 129)	PP (*n* = 81)	Asbestosis (*n* = 31)	MPM (*n* = 15)
Age, y median (IQR)	67 (8)	66 (8)	67 (7)	68 (10)	73 (16)^#^	66 (15)^●^
Gender						
Male, *n* (%)	44 (62.0)	47 (27.7)^*∗*^	21 (16.3)	33 (40.7)	8 (25.8)^*∗*^	7 (46.7)
Female, *n* (%)	27 (38.0)	123 (72.4)	108 (83.7)	48 (59.3)	23 (74.2)	8 (53.3)
Smoker, *n* (%)	10 (14.5)	16 (9.9)	7 (5.6)	13 (17.1)	1 (3.3)	4 (26.7)
Drinker, *n* (%)	5 (7.3)	9 (5.6)	5 (4.0)	6 (7.9)	0 (0.0)	4 (26.7)
Potential exposure level, mg/m^3^-years (median (IQR))	NA	17.7 (31.9)	70.8 (83.9)^#^	50.2 (77.0) ^#▲^	11.9 (83.3)^#▲^	11.0 (13.1)^#^
Exposure duration, y (median (IQR))	NA	5 (3.0)	14 (5.0)^#^	10 (8.5) ^#▲^	10 (10.0)^#▲^	14 (12.5)^▲^

PP: pleural plague; AE: asbestos-exposed; MPM: malignant pleural mesothelioma; IQR: interquartile range; NA: not applicable; Y: years.

*∗* versus control group, *P* < 0.05; # versus AE < 10 Y group, *P* < 0.05; ▲ versus AE ≥ 10 Y group, *P* < 0.05; ● versus asbestosis group, *P* < 0.05.

**Table 2:** Factors influencing serum levels of HMGB1 in univariable and multiple linear regression.

Predictor variables^*∗*^	Univariable analysis			Multivariable analysis^#^		
	*b*	SE	*P* value	*b*	SE	*P* value
Age	−0.001	0.002	0.66			
Gender (female/male)	0.05	0.03	0.07			
Smoking (nonsmoker/smoker)	−0.07	0.04	0.08			
Drinking (nondrinker/drinker)	−0.05	0.05	0.35			
PP group versus control group	0.20	0.04	0.0001	0.20	0.04	<0.0001
AE < 10 y group versus control group	0.23	0.04	<0.0001	0.23	0.04	<0.0001
AE ≥ 10 y group versus control group	0.22	0.04	<0.0001	0.22	0.04	<0.0001
Asbestosis group versus control group	0.44	0.06	<0.0001	0.44	0.06	<0.0001
MPM group versus control group	0.48	0.07	<0.0001	0.48	0.07	<0.0001

^*∗*^The dependent variable was natural logarithm form; ^#^estimated with forward stepwise method; *b*: regression coefficient; SE: standard error.

**Table 3:** AUC and cut-off value of HMGB1 comparing healthy controls, AE, and ARDs.

	AUC (95% CI)	*P* value	Cut-off (ng/ml)	Sensitivity (%)	Specificity (%)
MPM versus healthy	0.94 (0.89~0.99)	<0.0001	52.16	100	80.28
Asbestosis versus healthy	0.88 (0.82~0.95)	<0.0001	47.11	93.55	70.42
MPM versus AE < 10 Y	0.81 (0.73~0.90)	<0.0001	52.29	100	57.65
MPM versus AE ≥ 10 Y	0.80 (0.72~0.89)	0.0001	52.39	100	57.36
Asbestosis versus AE < 10 Y	0.74 (0.66~0.83)	<0.0001	58.43	61.29	77.06
Asbestosis versus AE ≥ 10 Y	0.74 (0.64~0.83)	<0.0001	56.48	64.52	72.09
PP versus healthy	0.68 (0.60~0.77)	0.0001	47.01	59.26	70.42
MPM versus asbestosis	0.56 (0.39~0.73)	0.5043	51.64	100	29.03

AUC: area under the curve; CI: confidence interval; AE: asbestos-exposed subjects; ARDs: asbestos-related diseases.

**Table 4:** Relationship between HMGB1 and MMPs covariates in study groups.

Group	Coefficient	(HMGB1, MMP2)	(HMGB1, MMP9)	(MMP2, MMP9)
Healthy	*r*_*s*_	0.1998	0.2580	0.586
	*P*	0.0949	**0.0298**	**<0.001**
AE < 10 Y	*r*_*s*_	0.2215	0.2721	0.345
	*P*	**0.0037**	**0.0003**	**<0.001**
AE ≥ 10 Y	*r*_*s*_	0.2345	0.1456	0.269
	*P*	**0.0075**	0.0997	**0.002**
PP	*r*_*s*_	0.0677	0.0099	0.494
	*P*	0.5483	0.9303	**<0.001**
Asbestosis	*r*_*s*_	−0.0343	−0.1347	0.018
	*P*	0.8548	0.4701	0.925
MPM	*r*_*s*_	0.0821	0.3464	0.725
	*P*	0.7710	0.2059	**0.002**

AE: asbestos-exposed; PP: pleural plaques; Y: years; *r*_*s*_: Spearman's rank correlation coefficient.
