# Supplementary material for: Serum HMGB1 as a Potential Biomarker for Patients with Asbestos-Related Diseases
Source: Dis Markers. 2017 Mar 1;2017:5756102. doi: 10.1155/2017/5756102 (PMC5350493; doi:10.1155/2017/5756102)
Supplement: Supplementary file 1 — Figure S1: MMP2 levels in serum from individuals with pleural plaques (PP), asbestosis, MPM, exposed to asbestos, and healthy controls. Figure S2: MMP9 levels in serum from individuals with pleural plaques (PP), asbestosis, MPM, exposed to asbestos, and healthy controls. [file 5756102.f1.pdf]

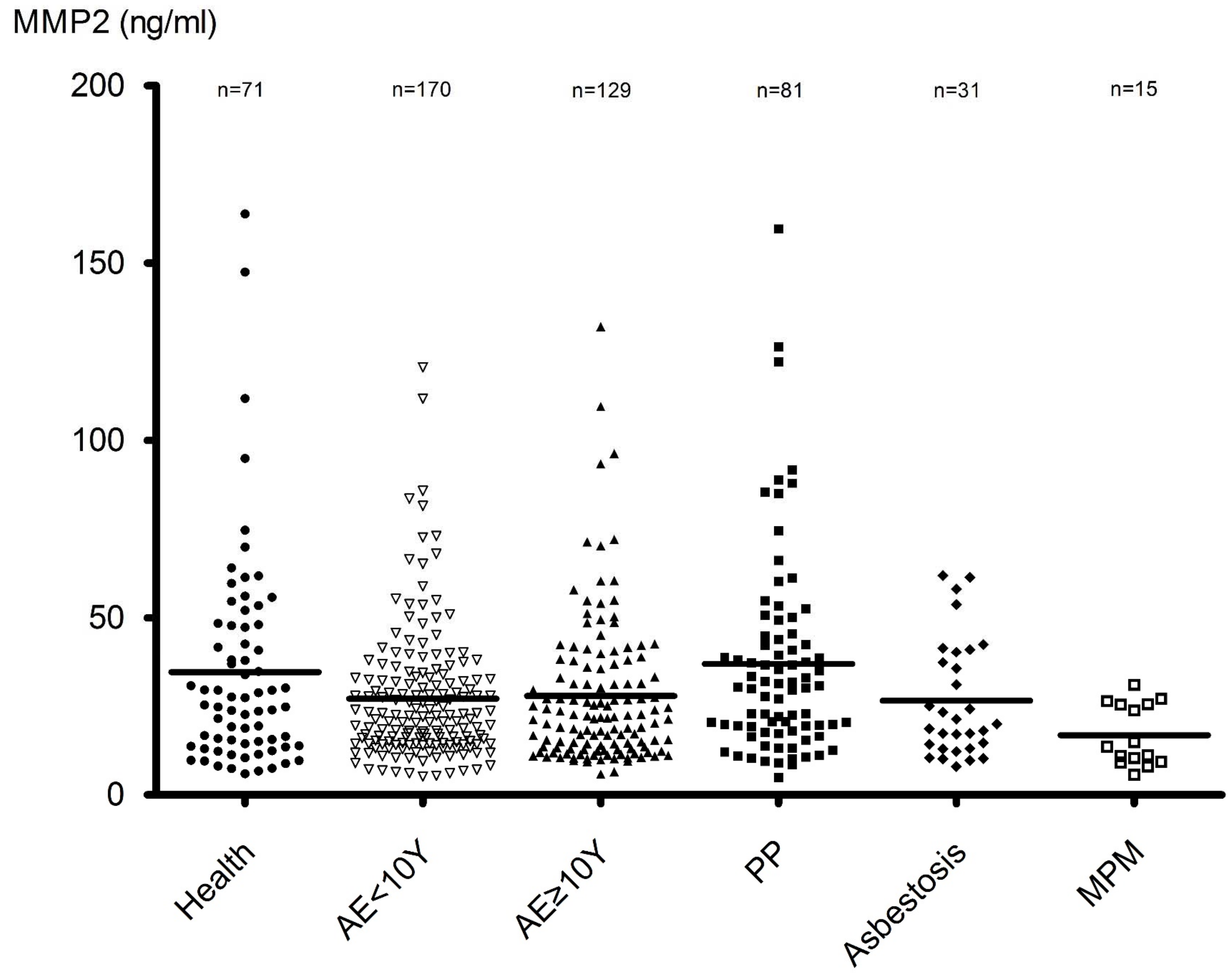

Figure S1. MMP2 levels in serum from individuals with pleural plaques (PP), asbestosis, MPM, exposed to asbestos and healthy controls. ELISAs shown were performed in parallel and blindly. Bars show the median of MMP2 levels.

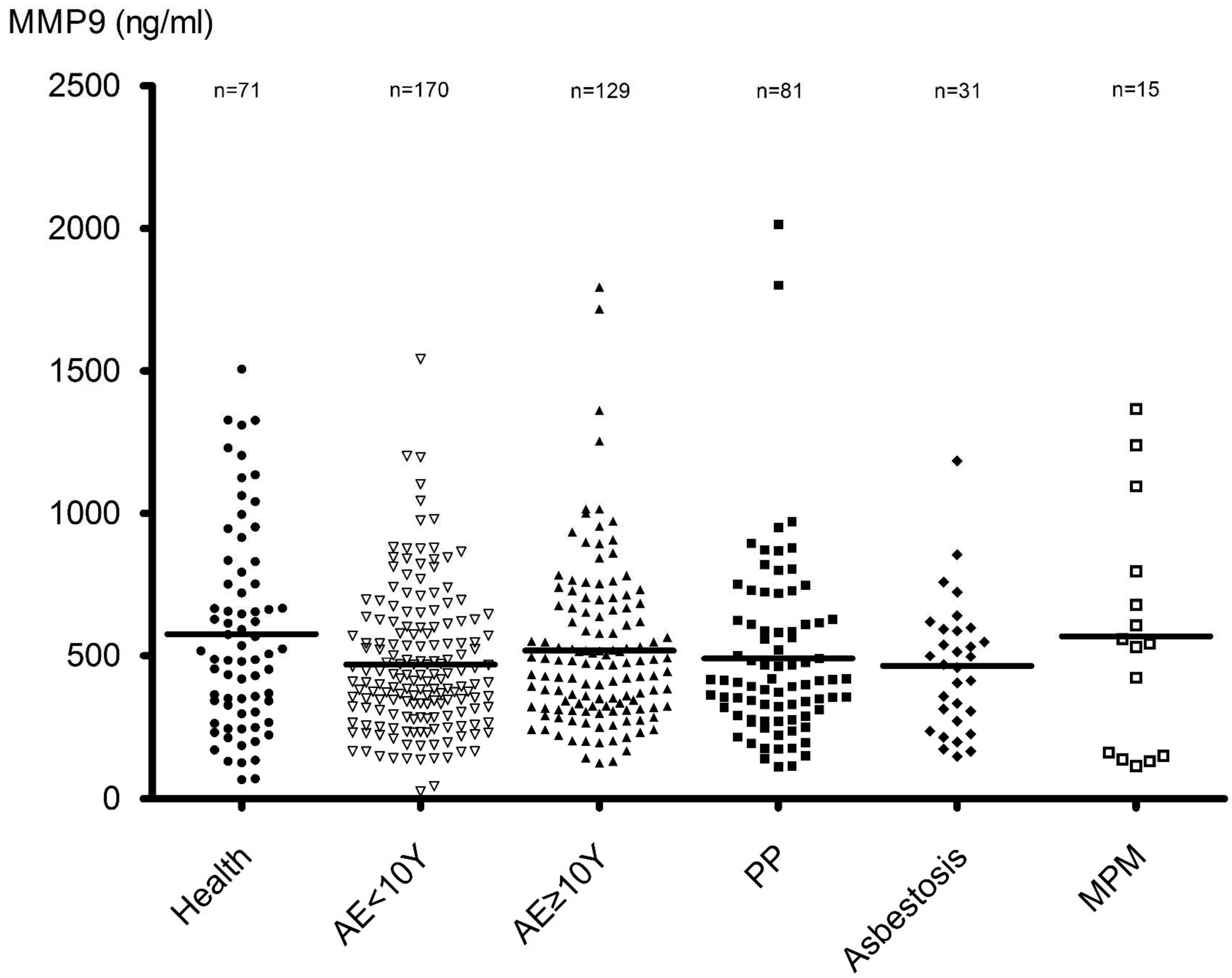

Figure S2. MMP9 levels in serum from individuals with pleural plaques (PP), asbestosis, MPM, exposed to asbestos and healthy controls. ELISAs shown were performed in parallel and blindly. Bars show the median of MMP9 levels.
